# RGS12 is required for the maintenance of mitochondrial function during skeletal development

**DOI:** 10.1038/s41421-020-00190-w

**Published:** 2020-09-01

**Authors:** Gongsheng Yuan, Shuting Yang, Min Liu, Shuying Yang

**Affiliations:** 1 Department of Basic and Translational Sciences, University of Pennsylvania, School of Dental Medicine, Philadelphia, PA USA; 2 The Penn Center for Musculoskeletal Disorders, University of Pennsylvania, School of Medicine, Philadelphia, PA USA; 3 Center for Innovation & Precision Dentistry, University of Pennsylvania, School of Dental Medicine, School of Engineering and Applied Sciences, Philadelphia, PA USA

**Keywords:** Mechanisms of disease, Ageing

## Abstract

Mitochondrial morphology and function are crucial for tissue homeostasis, such as for skeletal development, but the cellular and molecular mechanisms remain unclear. Here, we provide evidence that regulator of G-protein signaling 12 (RGS12) is present in the mitochondria of primary chondrocytes and cartilage tissues. Deletion of RGS12 in type II collagen-positive cells led to a significant decrease in mitochondrial number, membrane potential, and oxidative phosphorylation function. Mechanistically, RGS12 promoted the function of ATP5A as an enhancer of tyrosine phosphorylation. Mice with RGS12 deficiency in the chondrocyte lineage showed serious body retardation, decreased bone mass, and chondrocyte apoptosis due to the defective activity of ATP synthase. To our knowledge, this is the first report that RGS12 is required for maintaining the function of mitochondria, which may allow it to orchestrate responses to cellular homeostasis.

## Introduction

Endochondral ossification, an important process in skeletal growth, is highly dependent on the correct functioning of growth plate chondrocytes^1^. The endochondral bone forms through the continuing replacement of the cartilage matrix by calcified bone at the growth plate. In addition, apoptosis in cartilage is an important mechanism during the repair process in pathological conditions, such as arthritis, fracture, and cancer^2^. At the chondro-osseous junction, some hypertrophic chondrocytes undergo apoptosis/damage and allow the invasion of blood vessels delivering cells that continuously replace cartilage^3^. Growth plate cartilage is a highly metabolic tissue adapted to generate energy for bone growth^4^. Chondrocyte mitochondria in the growth plate demonstrated the same function as mitochondria in other tissues^5^. The increase in the mitochondrial protein fraction and the decrease in mitochondrial volume observed in reserve cartilage compared to the hypertrophic zone indicates that resident chondrocytes change their energy status^6^.

Mitochondria are critical controllers of cell function that have key roles in multiple bone diseases^7^. Electrons from hydrogen are passed from mitochondrial complexes I and II to complex III via the mobile electron carrier coenzyme Q10 and then travel from complex III–IV. Finally, complex V (ATP synthase) produces ATP from ADP and inorganic phosphate^8^. During the process of oxidative phosphorylation, mitochondria utilize oxygen not only to produce ATP from organic fuel molecules but also produce reactive oxygen species (ROS)^9^. As major locations of ROS generation, mitochondria are prone to apoptosis, oxidative damage and stress. Changes in mitochondrial morphology, distribution, and dynamics can lead to downstream physical, environmental, and genetic cues^10^. Additionally, mitochondrial diseases are mostly caused by impairment of oxidative phosphorylation (OXPHOS) or other energy metabolic defects^11^. Children with mitochondrial diseases caused by mitochondrial respiratory chain dysfunction often present with short stature, growth retardation, and abnormal bone development, indicating that the mitochondrial respiratory chain may be essential for cartilage-mediated skeletal growth^12,13^.

Mitochondrial complex V is a critical enzyme in the final steps of the mitochondrial respiratory chain. In living organisms, ATP synthesis accounts for most of the cellular ATP yield required to drive the many energy-consuming reactions and processes of the organism^14^. Complex V is a multisubunit enzyme complex containing F0 and F1 domains. The membrane-embedded F0 domain is a proton channel and consists of 10 subunits. The catalytic domain F1 is composed of five domains and a loosely attached protein IF1. The F1 domain converts ADP to ATP by using the electrochemical gradient, which consists of the final step of oxidative phosphorylation^15^. ATP5A1, also known ATP Synthase F1 Subunit Alpha, is a nuclear gene encoding a mitochondrial protein and a mitochondrial membrane ATP synthase, which is a crucial part of complex V. In humans, an ATP5A1 mutation leads to fatal neonatal mitochondrial encephalopathy, and complementation of the ATP5A1 defect restores the amount and activity of complex V^16^. Moreover, ATP5A1 is the most frequently affected subunit in tumors and benign prostate tissues^17^.

Regulator of G-protein signaling (RGS) proteins are a family of proteins that are involved in apoptosis and ROS regulation^18–20^. To date, several members of the RGS family of proteins have been isolated^21^, and specific RGS proteins have been reported to regulate specific signaling pathways^22^. Among them, RGS12 is the largest multidomain protein and a GTPase-activating protein for Gαi subunits^23,24^. In addition to a central RGS domain, RGS12 contains a PDZ (PSD-95/discslarge/ZO-1 homology) domain, a PTB (phosphotyrosine-binding) domain, a tandem repeat of Ras-binding domain and a GoLoco domain^24^. Our previous studies found that RGS12 is highly expressed in bone cells and regulates the function of osteoblasts and osteoclasts^19,25^. Endochondral bone growth is critically driven by the transformation of cartilage into bone tissue as a result of cell proliferation, differentiation, and apoptosis within the growth plate. Mitochondria in the growth plate play a critical role in the regulation of endochondral bone formation, matrix calcification, and apoptosis^5,26,27^. Whether RGS12 is involved in mitochondrial metabolism and endogenous ossification remains unknown.

For the first time, we found that RGS12 is expressed in mitochondria and plays a crucial role in regulating ATP5A and mitochondrial function in cartilage tissue and chondrocytes. Our data demonstrate that RGS12 is a critical activator of phospho-ATP5A, which in turn protects chondrocytes from apoptosis and osteogenesis disorders.

## Results

### RGS12 is expressed in chondrocyte mitochondria, cytoplasm, and nucleus

**Fig. 1:**
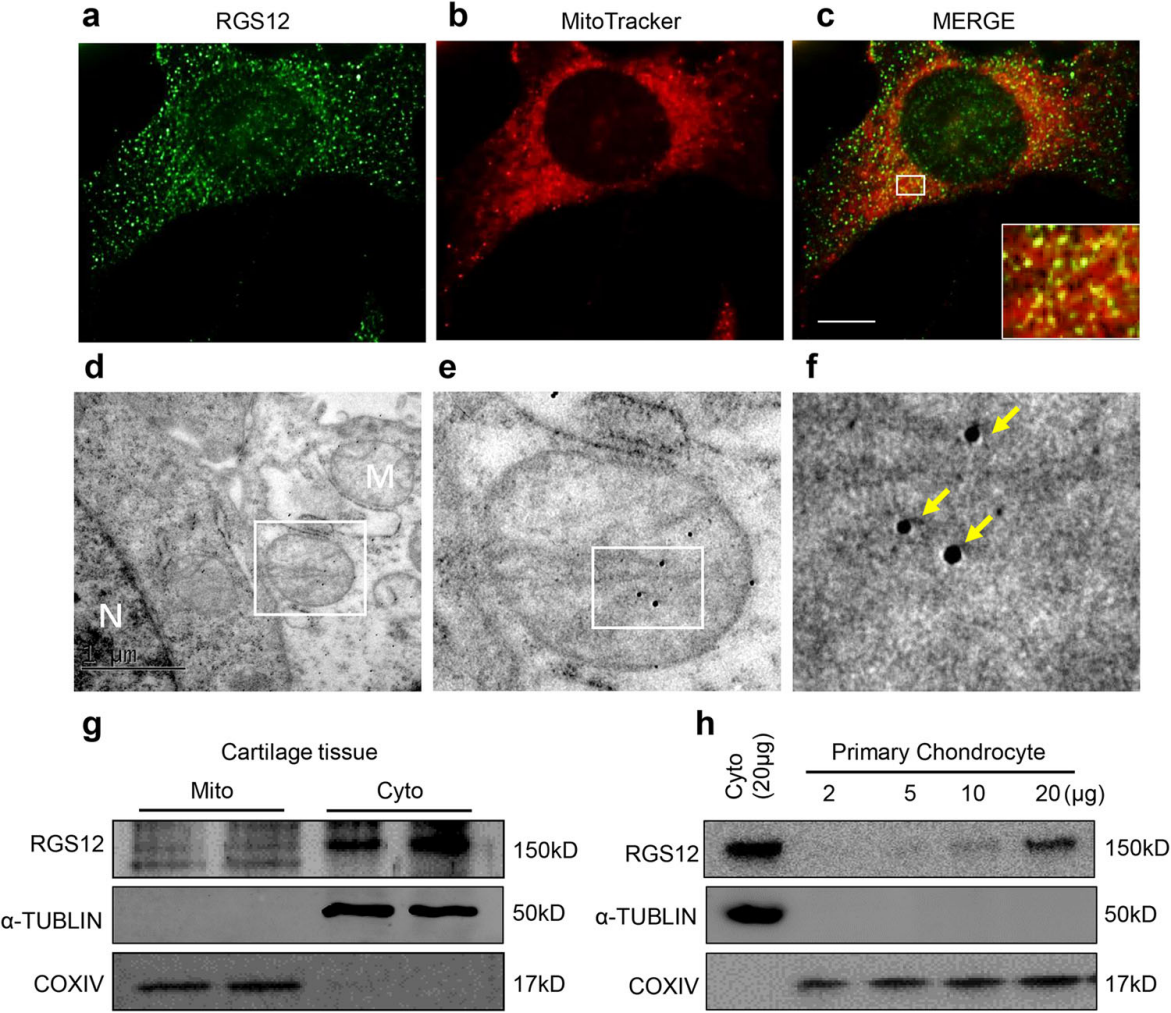
RGS12 is located within the mitochondria. **a–c** Representative immunostaining of RGS12 (**a**) (green), MitoTracker (**b**) (red) protein in primary chondrocytes. Representative merge image of RGS12 (green) and MitoTracker (red) in primary chondrocytes (Scale bar, 5 μm) (**c**). **d–f** Immunogold electron microscopy (post embedding) method to label RGS12 in primary mouse chondrocytes with nanogold particles. Localization of RGS12 detected with anti-RGS12 in mitochondria (yellow arrows). M Mitochondria, N Nucleus. **g** Mouse mitochondrial (Mito) and cytoplasmic (Cyto) extracts from cartilage tissues were separated by SDS-PAGE. The blots were probed with anti-RGS12, α-TUBLIN, and COXIV. **h** Increasing amounts of chondrocyte mitochondria probed for RGS12, α-TUBLIN, and COXIV.

RGS12 has been identified as a protein related to cell proliferation, differentiation, oxidative phosphorylation, ROS production, and apoptosis^19,25^. These observations indicate that RGS12 may play a key role in mitochondria. To examine the cellular localization of RGS12, RGS12 was immunostained with anti-RGS12 antibody in primary chondrocytes (Fig. 1a), and mitochondria were visualized using MitoTracker (Fig. 1b). RGS12 was distributed homogeneously in the cytoplasm (69.38%) and nuclear regions (30.62%) of chondrocytes (Supplementary Fig. S1). A portion of RGS12 colocalized with MitoTracker in the perinuclear mitochondrial regions (Fig. 1c). To further confirm the RGS12 location, we then carried out transmission electron microscopy (TEM) analysis of mouse chondrocytes to determine the precise location of RGS12. We observed RGS12 positivity in the mitochondria, cytoplasm, and nucleus, which is similar to the IF expression pattern (Fig. 1d–f). To address the role of RGS12 in chondrocytes, we isolated mitochondrial and cytoplasmic fractions from mouse cartilage (1-month-old mice) and used immunoblotting to detect the expression level of RGS12. RGS12 was present in the mitochondrial and cytoplasmic fractions (Fig. 1g). The amount of RGS12 in the mitochondria was approximately one-tenth of that in the cytosol (Fig. 1g). The blots were also probed for α-TUBLIN as a cytoplasmic marker and COXIV as a mitochondrial marker. To confirm that RGS12 in chondrocyte mitochondria did not come from a contamination by cytoplasmic RGS12, purified mitochondria from primary chondrocytes were used for the expression of RGS12, α-TUBLIN, and COXIV (Fig. 1h). These results showed that RGS12 was expressed in a dose-dependent pattern along with the mitochondrial amount from primary chondrocytes.

### RGS12 regulates tyrosine phosphorylation of ATP5A

**Fig. 2:**
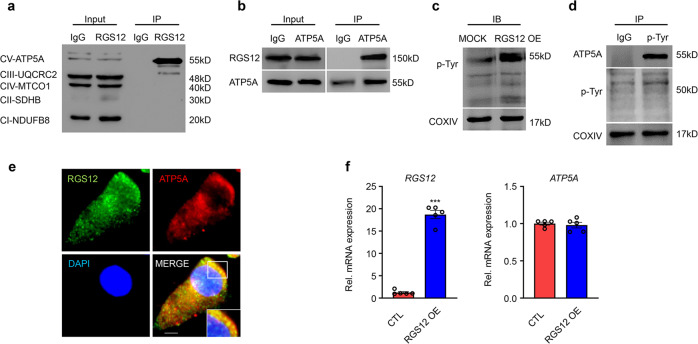
RGS12 regulates tyrosine phosphorylation of ATP5A. **a** The cell lysates of mouse chondrocytes were incubated with anti-RGS12 or control immunoglobulin G (IgG) antibodies, and bound protein was examined by western blotting (WB) with the OXPHOS antibodies, as indicated. Note only ATP5A directly associates with RGS12. **b** ATP5A associates with mitochondrial RGS12. Antibody to ATP5A or a nonspecific isotype-matched IgG were incubated with chondrocyte mitochondrial extracts. Immunoprecipitates were resolved on SDS-PAGE and probed for RGS12. **c** RGS12 affects mitochondrial Phos-Tyrosine (p-Tyr) expression. ATDC5 cells were transfected with pcDNA3.1 (MOCK) and pcDNA3.1-RGS12 plasmids (RGS12 Overexpression; RGS12 OE) for 48 h and used to measure mitochondrial Phos-Tyr (p-Tyr) protein level by immunoblotting. **d** Phos-Tyr (p-Tyr) interacts with ATP5A in mitochondria (Co-IP assay). The mitochondrial lysates were incubated with anti-Phos-Tyr (p-Tyr) or control immunoglobulin G (IgG) antibodies, and bound protein was examined by western blotting (WB) as indicated. **e** Immunofluorescence showing that RGS12 and ATP5A show colocalization in chondrocytes. Scale bar, 5 µm. Representative individual and overlaid images are shown. **f** Expression levels of *RGS12* and *ATP5A* in Control (CTL) and RGS12 overexpression (RGS12 OE) chondrocytes were determined by quantitative PCR. Note that *ATP5A* was not significantly changed in RGS12 OE chondrocytes. Values are means ± SEM. *n* = 5, ***P < 0.001.

In a search for possible targets for RGS12 in mitochondria, we tested OXPHOS, a core mitochondrial protein essential for the maintenance of the cell steady-state and regulation of ROS^28^. First, we analyzed whether various OXPHOS proteins, such as ATP5A, UQCRC2, MTCO1, SDHB, and NDUFB8, are associated with RGS12 in mouse chondrocytes. The results unequivocally revealed that RGS12 binds with ATP5A (Fig. 2a) but does not bind with UQCRC2, MTCO1, SDHB, or NDUFB8 (Fig. 2a), emphasizing the specificity of RGS12-mediated mitochondrial functions. We then detected RGS12 in immunoprecipitates of mitochondrial extracts from chondrocytes using an ATP5A-specific monoclonal antibody. As expected, RGS12 was immunoprecipitated with the ATP5A antibody, indicating the presence of mitochondrial RGS12 (Fig. 2b). Phosphorylation of tyrosine residues in ATP synthase subunits is critical and highly conserved across mammalian species^29^. Interestingly, we found that overexpression of RGS12 (RGS12 OE) could enhance mitochondrial phosphorylated tyrosine (p-Tyr) in chondrocytes (Fig. 2c). We further found that anti-Tyr(P) IP fractions from mitochondrial lysates contained ATP5A (Fig. 2d), suggesting that p-Tyr of ATP5A associates with RGS12. Additionally, RGS12 and ATP5A showed colocalization in primary chondrocytes by immunofluorescence (Fig. 2e). However, overexpression of RGS12 did not affect ATP5A mRNA transcription (Fig. 2f).

### Knockdown of RGS12 in chondrocytes leads to mitochondrial loss and ATP deficit

**Fig. 3:**
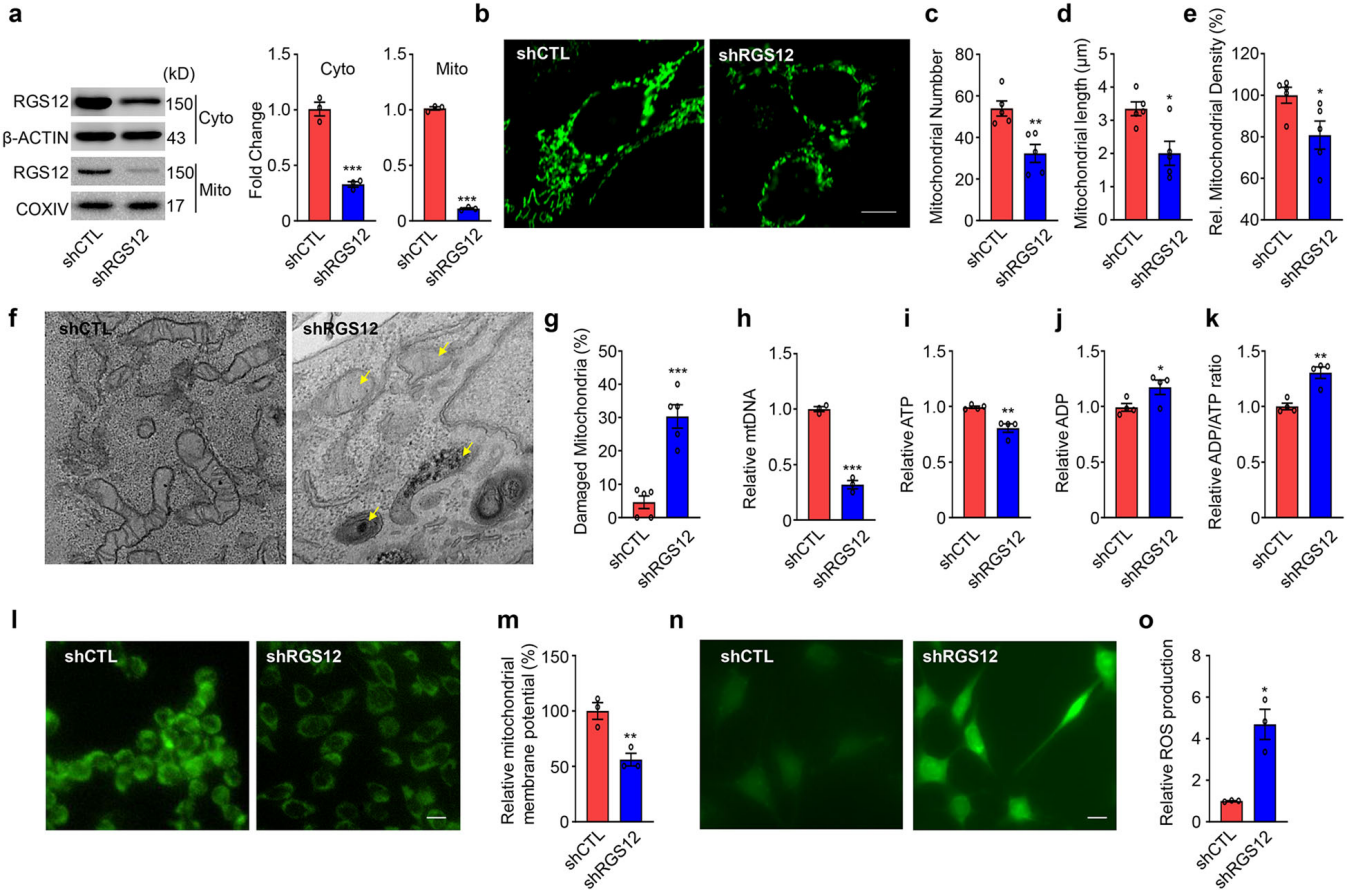
Knockdown of RGS12 leads to mitochondrial loss and ATP deficit. **a** Western blot analysis of RGS12 expression in cytoplasm (Cyto) and mitochondria (Mito) when transfected with scramble control shRNA (shCTL) or shRGS12 in primary chondrocytes. Data are expressed as mean ± SEM (*n* = 3, ***P < 0.001). **b** Representative images of MitoTracker green staining. Quantitative measurement of (**b**), mitochondrial number (**c**), mitochondrial length (**d**) and mitochondrial density (**e**) in shCTL and shRGS12 groups using NIH Image J software. *n* = 5, **P < 0.01, *P < 0.05. (**f**) Representative chondrocytes transmission electron microscopy (TEM) images from shCTL and shRGS12-treated chondrocytes (yellow arrows showed the swollen and damaged mitochondria). **g** Quantification of the damaged mitochondria in **f**. **h** Mitochondrial DNA (mtDNA) copy number in chondrocytes as determined by PCR. Note that mtDNA copy number was decreased in shRGS12-treated chondrocytes compared with shCTL. Values are means ± SEM. *n* = 3. Relative ATP level (**i**), ADP level (**j**), and ADP/ATP ratio (**k**) for each separate group are shown in **i–k**. **l** Rhodamine 123 stained chondrocytes showing bright and typical mitochondrial network morphology but low intensity in shRGS12-treated cells. **m** Relative mitochondrial membrane potential. **n** Intracellular ROS production was increased in shRGS12-treated chondrocytes relative to shCTL chondrocytes. **o** Relative ROS quantification of **n**. Data are means ± SEM. *P < 0.05, **P < 0.01.

To explore whether the knockdown of RGS12 affects mitochondrial function, primary chondrocytes were treated with scramble control shRNA (shCTL) or shRGS12 (Fig. 3a). We found that the protein expression levels of RGS12 in the cytoplasm and mitochondria from primary chondrocytes transfected with shRGS12 were both significantly reduced compared with those in the shCTL group (Fig. 3a). We then performed MitoTracker staining (Fig. 3b). The results showed that the number of mitochondria was significantly decreased in the shRGS12 group (Fig. 3c), and the length of mitochondria and relative mitochondrial density were significantly reduced in chondrocytes treated with shRGS12 (Fig. 3d, e). Analysis of mitochondrial morphology by TEM showed that the number of mitochondria decreased, but the damaged mitochondria significantly increased in the shRGS12 group compared to the control (Fig. 3f, g). As expected, chondrocytes in the shRGS12 group exhibited significantly lower mtDNA (mitochondrial DNA) copy numbers than those in the control group (Fig. 3h). To further analyze the effect of RGS12 on the bioenergetic function of chondrocyte mitochondria, we compared the relative ATP level, the relative ADP level and the ADP/ATP ratio in chondrocytes from the shCTL and shRGS12 groups (Fig. 3i–k). The results showed that knockdown of RGS12 decreased the relative ATP level (Fig. 3i) but significantly increased the relative ADP level (Fig. 3j) and ADP/ATP ratio (Fig. 3k). Moreover, to examine the mitochondrial membrane potential (MMP), we labeled chondrocyte mitochondria with rhodamine 123, a cationic fluorescent dye that can label functional mitochondria without cytotoxic effects^30^. The results showed that mitochondria in shRGS12-treated chondrocytes had lower rhodamine 123 fluorescence than those in the control group (Fig. 3l, m), demonstrating that RGS12 is required for the maintenance of MMP. To further assess mitochondrial function, we measured basal mitochondrial ROS production using DCFDA, which has been used to measure intracellular ROS production^31^. Chondrocytes treated with shRGS12 exhibited increased levels of mitochondrial ROS production in comparison with control cells (Fig. 3n, o).

### Cartilage-specific RGS12 KO mice display postnatal growth retardation, shortening of long bones, and growth plate defects

**Fig. 4:**
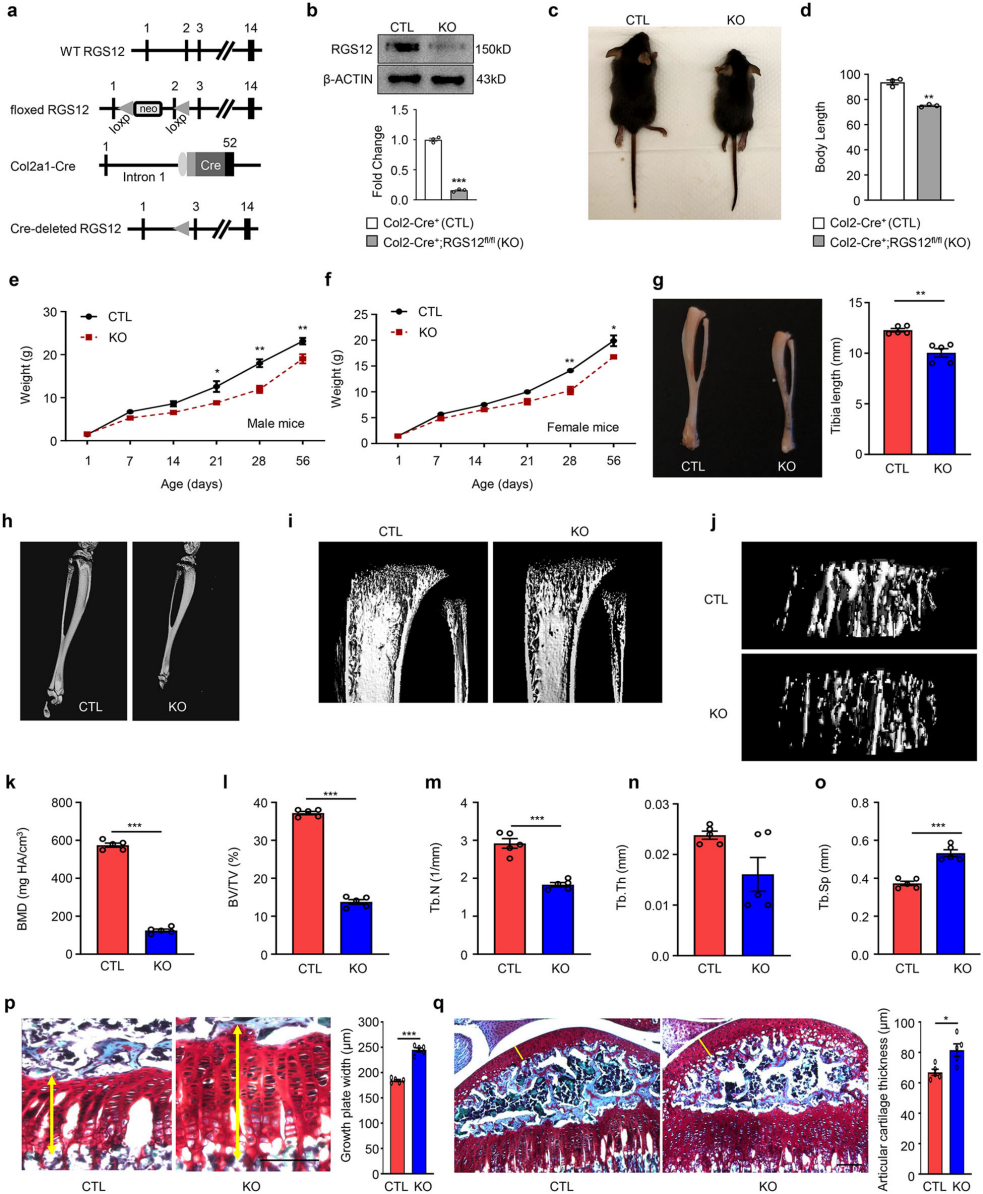
Cartilage-specific RGS12 KO mice display postnatal growth retardation, shortening of long bones and growth plate defects. **a** Organization of the wild-type RGS12 allele, the floxed RGS12 targeting construct, the Col2a1-cre transgene, and the recombinant RGS12 allele. Exons are indicated by black boxes and numbered. neo: neomycin resistance gene, loxP: loxP sites, Cre: Cre recombinase coding sequence. **b** Cartilage derived from RGS12 KO (RGS12-knockout, Col2-Cre^+^;RGS12^fl/fl^) or CTL (Control, Col2-Cre^+^) were immunoblotted with an antibody against mouse RGS12. β-ACTIN was used as a loading control. **c** One-month-old RGS12 KO mice show growth retardation. **d** Quantitative data showed body size reduction was detected in RGS12 KO mice compared to CTL littermates as described in **c**. **e**, **f** The postnatal growth curve shows a lower weight in male and female KO animals. The highest weight difference in RGS12 KO mice compared to CTL mice was observed at the age of 1 month. *n* = 5, *P* < 0.05, **P < 0.01. **g** Images of tibias from CTL and RGS12 KO mice at age 1 month (left). Quantification of length of tibia (right). Red bar represents CTL mice and blue bar represents RGS12 KO mice, and (*n* = 5). **h**–**j** Representative μCT images of tibias from a 1-month-old male RGS12 KO mouse and a CTL littermate. **k**–**o** Quantitative analysis of μCT data of 1-month-old tibias reveals a lower BMD (**k**), BV/TV (**l**), and a reduced number of bone trabeculae (**m**) together with an increased trabecular separation (**o**) in RGS12 KO animals compared to Col2-Cre^+^ controls. The trabecular thickness (**n**) displays no statistically significant difference between the genotypes. **p** Safranin O staining of tibial proximal growth plates. Arrows indicate increased growth plate width in RGS12 KO mice (left). Width measurements of total growth plate (right). *n* = 5, ***P < 0.001. **q** Knockout of RGS12 increased the thickness of the articular cartilage in 4-week-old mice. Left: sagittal sections at the level of the mid-medial tibial plateau are shown (yellow line). Right: measurements of cartilage thickness of the RGS12 KO mice. Data are presented as means ± SEM. *P < 0.05.

To define the role of RGS12 in skeletal growth, cartilage-specific RGS12 KO mice were generated by mating RGS12 floxed mice with Col2a-Cre transgenic mice expressing the cre recombinase under the control of the alpha1 type Collagen II (Col2) promoter, which is active predominantly in chondrocytes. In this setting, the cre recombinase removed exon 2 of the floxed RGS12 gene (Fig. 4a) in Col2^+^ cells. Loss of RGS12 protein from cartilage was confirmed by immunoblotting of tibial cartilage of RGS12 mutant newborn mice (Fig. 4b). The cartilage-specific RGS12 KO (Col2-Cre^+^;RGS12^fl/fl^) mice were viable and fertile but developed postnatal growth retardation (Fig. 4c, d). The weights of control (CTL, Col2-Cre^+^) males and females and their RGS12-knockout (KO, Col2-Cre^+^;RGS12^fl/fl^) littermates were determined at postnatal days 1, 7, 14, 21, 28 and 56 (Fig. 4e, f). The average weight of males showed a significant difference starting from day 21, whereas the females showed a significant difference starting from day 28 (Fig. 4e, f). By analyzing the long bones of CTL and RGS12 KO mice, we found a dramatic decrease in the tibia length of RGS12 KO mice compared to CTL mice at the age of 1 month (Fig. 4g). Micro-CT (μCT) results further showed that the bone length and trabecular bone mass of RGS12 KO mice (Fig. 4h–j) at the age of 1 month significantly decreased compared with those in CTL mice of the same age. Moreover, the trabecular BMD (Fig. 4k), BV/TV (bone volume over total volume) (Fig. 4l), the average trabecular number (Tb.N) (Fig. 4m) and the trabecular thickness (Tb.Th) (Fig. 4n) significantly decreased, while trabecular spacing (Tb.Sp) (Fig. 4o) increased in RGS12 KO mice. We then analyzed the trabecular bone mass of 12-week-old mice (Supplementary Fig. S2). However, of the investigated parameters, only BV/TV and the trabecular number showed a significant difference between RGS12 KO and CTL mice at the age of 12 weeks. We also analyzed the femoral cortical bone in 4-week- and 12-week-old mice (Supplementary Fig. S3). The results showed a significant decrease in femoral cortical bone volume and thickness in 4-week-old RGS12 KO mice but not 12-week-old KO mice, suggesting an early delay in postnatal bone growth peaking around the age of 1 month. By measuring the calvarial bone mass of RGS12 KO mice (4 weeks), we did not find a significant difference (Supplementary Fig. S4), indicating that RGS12 may not affect intramembranous ossification. Safranin O green staining of the hind limb sections from CTL and KO mice and further histomorphometry analysis showed that the width of growth plates (Fig. 4p) and the thickness of the articular cartilage (Fig. 4q) were increased in 4-week-old RGS12 KO animals. These results suggest a temporal delay in chondrocyte maturation in mutant animals.

### Absence of RGS12 in chondrocytes results in mitochondrial-dependent apoptosis

**Fig. 5:**
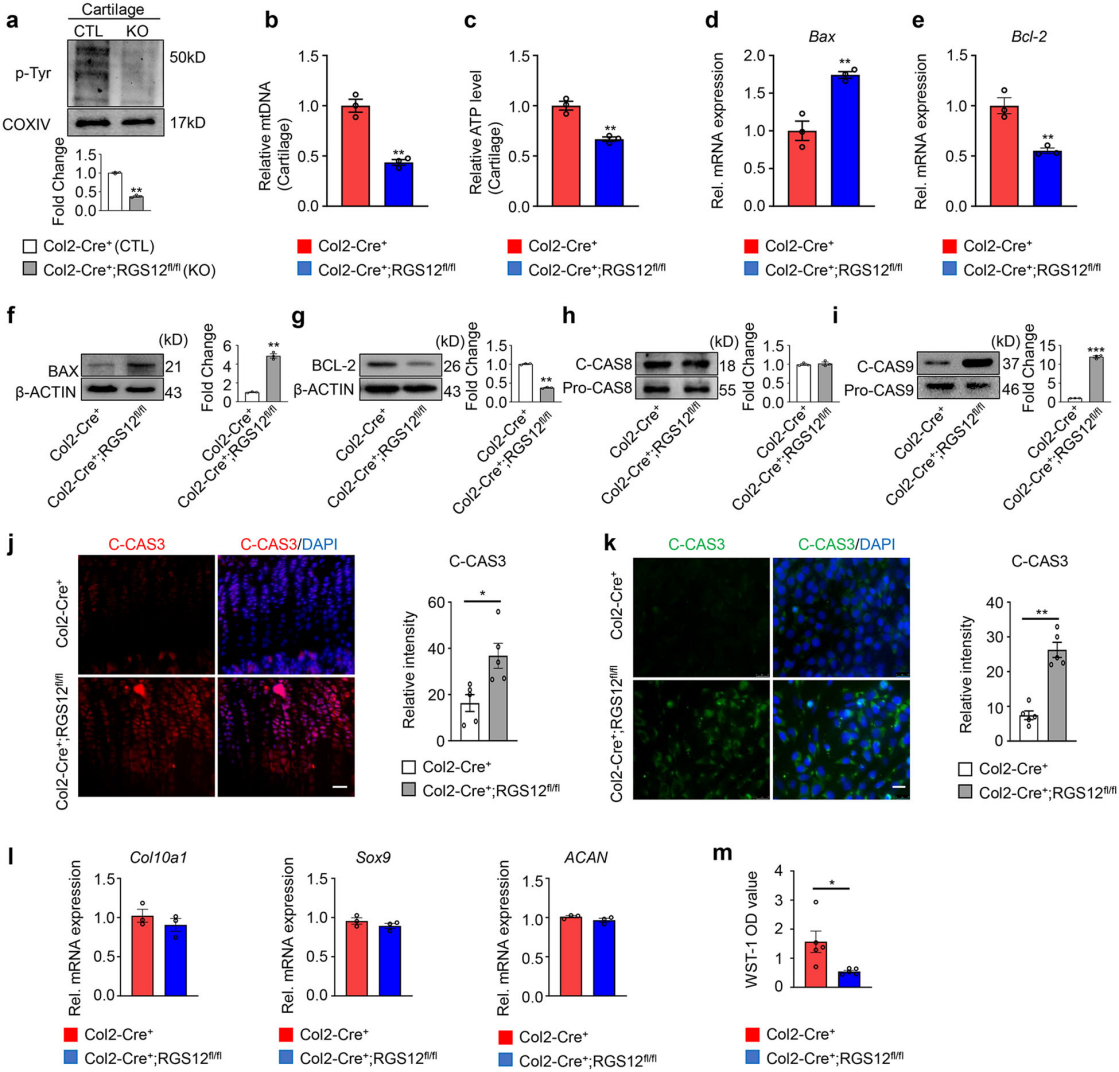
Absence of RGS12 in chondrocytes results in mitochondrial-dependent apoptosis. **a** Representative immunoblots for mitochondrial p-Tyr in cartilage from CTL(Col2-Cre^+^) and RGS12 KO (Col2-Cre^+^;RGS12^fl/fl^). Note that p-Tyr level in CTL mice was higher than that in KO mice. *n* = 3, *P < 0.05. **b** Mitochondrial DNA copy number in cartilage from Col2-Cre^+^ and Col2-Cre^+^;RGS12^fl/fl^ mice as determined by PCR. Values are means ± SEM. *n* = 3, **P < 0.01. **c** Relative ATP level in cartilage from Col2-Cre^+^ and Col2-Cre^+^;RGS12^fl/fl^ mice are shown. *n* = 3, **P < 0.01. **d**, **e** The relative gene expression l**e**vels of *Bax* and *Bcl-2*. The *t*-test showed significant differences between the two groups, *P < 0.05, **P < 0.01 versus Col2-Cre^+^. **f**–**i** Left panels show representative immunoblots for BAX (**f**), BCL-2 (**g**), C-CAS8 (cleaved-CASPASE-8) (**h**), Pro-CAS8 (pro-CASPASE-8) (**h**), C-CAS9 (cleaved-CASPASE-9) (**i**), and Pro-CAS9 (pro-CASEPASE9) (**i**) in cartilage from Col2-Cre^+^ and Col2-Cre^+^;RGS12^fl/fl^. Right panels show the quantitative date of relative intensity separately. Note that there was no significant change in relative C-CAS8 level. *n* = 3, **P < 0.01, ***P < 0.001. **j** Cleaved-CASPASE-3 (C-CAS3) immunohistochemistry to detect apoptosis cells within immature chondrocytes in growth plate from Col2-Cre^+^ and Col2-Cre^+^;RGS12^fl/fl^ mice tibia (left). Right panel shows the quantitative date of relative intensity in **j**. **k** Primary chondrocytes from Col2-Cre^+^ and Col2-Cre^+^;RGS12^fl/fl^ mice were stained with C-CAS3 antibody to detect the apoptosis level. Right panel shows the quantitative date of relative intensity in **k**. *n* = 3, *P < 0.05, **P < 0.01. Note that C-CAS3 level dramatically increased i*n* Col2-Cre^+^;RGS12^fl/fl^ chondrocytes. **l** Gene expression of *Col10a1*, *Sox9* and *ACAN* from cartilage of Col2-Cre^+^ and Col2-Cre^+^;RGS12^fl/fl^ mice. Note that there are no significance changes among these genes. *n* = 3, *P < 0.05. **m** Cell viability as measured by the WST-1 colorimetric assay for chondrocytes.

RGS12 can regulate p-Tyr and mitochondrial function (Figs. 2, 3). We first determined whether the mitochondrial number and function in cartilage from RGS12 KO mice changed. Our results showed that mitochondrial p-Tyr (Fig. 5a), mitochondrial number (Fig. 5b) and relative ATP (Fig. 5c) were decreased. Mitochondria play key roles in activating apoptosis^32^, and our data showed that the knockdown of ATP5A leads to apoptosis activation, as demonstrated by determining the expression of the *Bax/Bcl-2* genes (Supplementary Fig. S5). To confirm that apoptosis is also activated in RGS12 KO cartilage, we examined the mRNA levels of the apoptosis regulators *Bax* and *Bcl-2*. The results showed that the mRNA levels of the apoptotic *Bax* significantly increased (Fig. 5d), but those of the antiapoptotic gene *Bcl-2* significantly decreased (Fig. 5e), suggesting that the mitochondrial apoptotic process was activated in RGS12 KO mice. We then confirmed the mRNA data by performing immunoblotting and found the same results as the gene expression assay, which showed increased BAX (Fig. 5f) and decreased BCl-2 protein levels (Fig. 5g) in RGS12 KO mice.

Apoptosis has two classic signaling pathways: the extrinsic pathway and the intrinsic pathway. The extrinsic pathway is regulated by CASPASE-8, while the intrinsic pathway is mediated by CASPASE-9, and both pathways can trigger apoptosis through CASPASE-3^33^. To determine which pathway plays a role in RGS12 KO cartilage, we performed an immunoblot assay and found that activated CASPASE-8 (cleaved-CASPASE-8) did not show a significant change (Fig. 5h) in RGS12 KO cartilage, while cleaved-CASPASE-9 was dramatically elevated (Fig. 5i) in RGS12 KO cartilage compared with the CTL cartilage, suggesting that the intrinsic pathway (mitochondria-dependent pathway) plays a critical role in RGS12 KO-induced apoptosis. To further gain insight into the mechanism of the shortening of long bones and the increase in total growth plate length or hypertrophic zones, we determined whether abnormal apoptosis is activated in growth plates. Indeed, a higher number of chondrocytes undergoing apoptosis (highly expressing C-CAS3) was detected in the long bones of RGS12 KO males at 1 month of age (Fig. 5j) compared to CTL mice. Apoptotic cells were predominant throughout all regions of the growth plate compared to CTL tibia sections, which only had a limited number of apoptotic cells at the chondro-osseous junctions (Fig. 5j). Similarly, the number of apoptotic cells significantly increased in primary cultured chondrocytes isolated from the RGS12 KO group, which is similar to the in vivo data (Fig. 5k). We also found that the relative mRNA expression levels of chondrogenic-related genes, such as *collagen type 10 a1 (Col10a1)*, *Sox9*, and *ACAN*, were mildly decreased in RGS12 KO cartilage compared to CTL cartilage (Fig. 5l), suggesting that RGS12 may not regulate cartilage differentiation. The WST-1 assay for cell viability analysis also demonstrated that deletion of RGS12 in chondrocytes inhibited cell proliferation and viability (Fig. 5m).

### Restoring RGS12 expression reverses the abnormal bone and mitochondrial phenotype

**Fig. 6:**
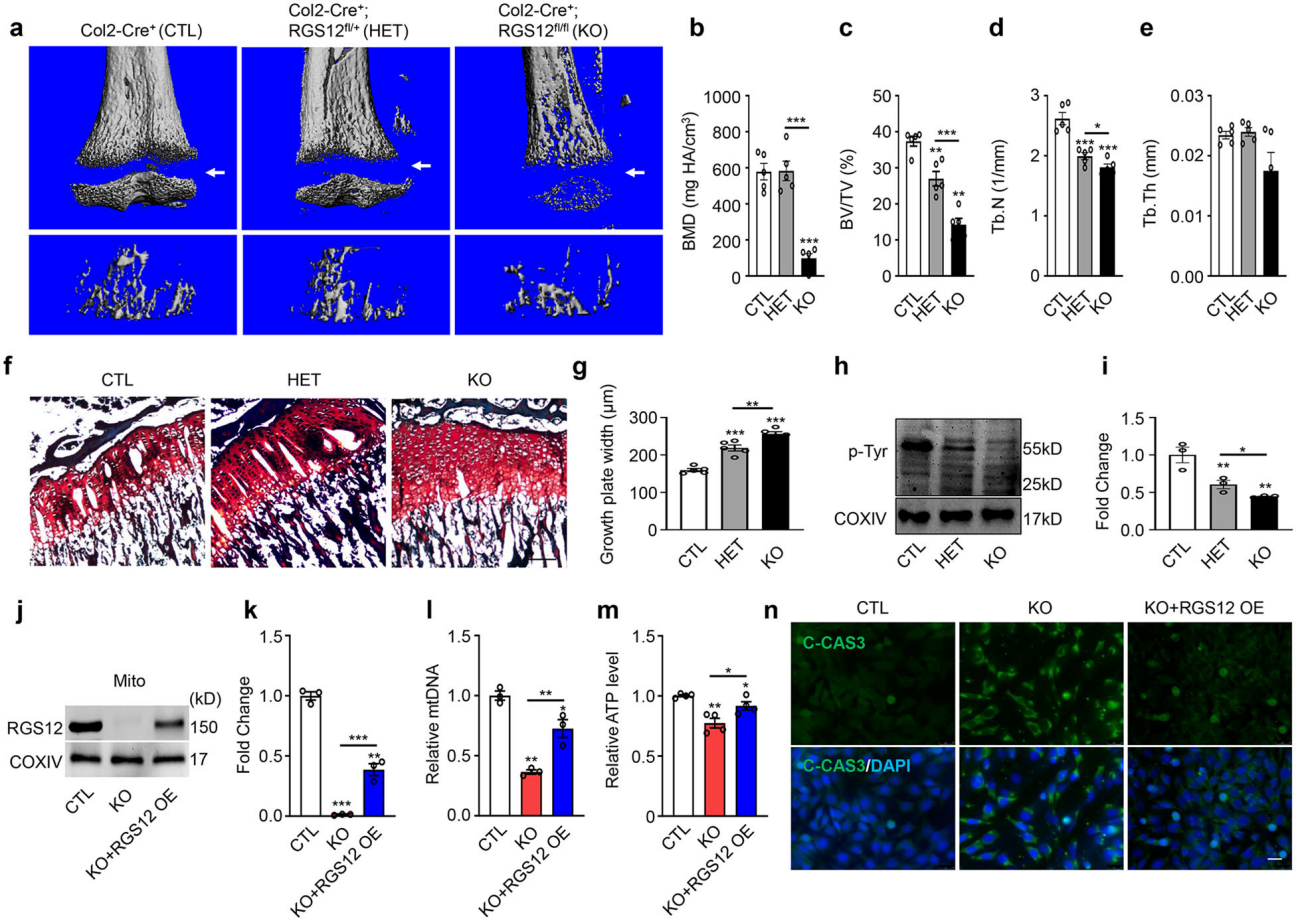
Restoring RGS12 expression reverses the abnormal bone and mitochondrial phenotype. **a** Representative 3D reconstructions of μCT images of tibias from a 1-month-old male CTL (Col2-Cre^+^), HET(Col2-Cre^+^;RGS12^fl/+^) and KO (Col2-Cre^+^;RGS12^fl/fl^) (*n* = 5) mouse indicate widened growth plates (white arrow) in HET and KO bones. One-month-old tibias of HET and KO mice also show less trabecular structures in metaphyseal bone areas. **b**–**e** Quantitative analysis of micro-CT data of tibias reveals BMD (**b**), BV/TV (**c**), trabecular number (**d**), and trabecular thickness (**e**) in CTL, RGS12 HET, and RGS12 KO mice. Note that RGS12 HET mice show an increased BMD, BV/TV and trabecular number compared to KO mice but show a decreased BV/TV and trabecular number compared to CTL mice. *n* = 5, *P < 0.05, **P < 0.01, ***P < 0.001. **f** Representative Safranin O stained sections of CTL, RGS12 HET and RGS12 KO mice at 1 month. **g** Quantitative analysis of growth plate length of CTL, RGS12 HET and RGS12 KO mice at 1 month. Note the differences in growth plate morphology between CTL, HET and KO at the age of 1 month. RGS12 HET partially rescued the RGS12 KO phenotype. **h** Representative immunoblots for mitochondrial p-Tyr in mouse cartilage. **i** Histogram depicts p-Tyr level normalized to COXIV in CTL, HET, and KO. Bar graph shows decreased p-Tyr level in HET and KO when compared to CTL. Note that p-Tyr level in HET mice was higher than that in KO mice. Unpaired Student’s *t*-test; *n* = 3, **P < 0.01, *P < 0.05. **j** Primary chondrocytes were extracted from CTL and KO mice, and one group of RGS12 KO cells were overexpressed with RGS12-Flag plasmids (3 μg) (KO + RGS12 OE). Western blot analysis of RGS12 in mitochondria from CTL, KO, and KO + RGS12 OE primary chondrocytes. Mito: mitochondria. **k** Quantitative analysis of the western blot results as shown in **j**. **l** Mitochondrial DNA copy number in primary chondrocytes as determined by PCR. Note that the mtDNA copy number of RGS12 OE in KO chondrocytes was higher than that in RGS12 KO chondrocytes. Values are means ± SEM. *n* = 3. **m** Relative ATP level in primary chondrocytes in the CTL, KO, and KO + RGS12 OE groups are shown. Data are presented as mean ± SEM. *P < 0.05, **P < 0.01. **n** Representative images illustrate active C-CASPASE-3 (C-CAS3) staining. Note that RGS12 OE reversed the apoptosis caused by the gene deletion.

To further characterize whether restoring RGS12 can promote osteogenesis in vivo, we first performed micro-CT analysis in RGS12 KO, RGS12 HET (heterozygotes, Col2-Cre^+^; RGS12^fl/+^), and control mice. Micro-CT images showed that the trabecular mass decreased and the gap between the tibial diaphysis and epiphysis was remarkably longer in 1-month-old RGS12 KO mice than in RGS12 HET and control mice, which suggests that the gain of RGS12 in vivo could reverse the abnormal bone phenotype (Fig. 6a). Moreover, RGS12 HET mice showed a reversed trabecular BMD (Fig. 6b), BV/TV (bone volume over total volume) ratio (Fig. 6c) and trabecular number (Tb.N) (Fig. 6d) but not trabecular thickness (Tb.Th) (Fig. 6e) compared to the RGS12 KO mice. As expected, the length of tibial growth plates in cartilage-specific RGS12 HET mice was also shorter than that in RGS12 KO mice, which was similarly evident by μCT (Fig. 6f, g). RGS12 regulates bone formation and mitochondrial function through the p-Tyr level. To further determine whether restoring RGS12 can promote p-Tyr, we extracted mitochondria from CTL, HET and KO mouse cartilage and found that RGS12 HET mice could reverse the mitochondrial p-Tyr level compared to RGS12 KO mice (Fig. 6h, i). To confirm that exogenous RGS12 could reverse the abnormal mitochondrial phenotype, we overexpressed RGS12 (RGS12 OE) in RGS12 KO primary chondrocytes and confirmed that RGS12 protein expression was restored in mitochondria by western blotting (Fig. 6j, k). We found that the overexpression of RGS12 reversed the mitochondrial numbers (Fig. 6l) and relative ATP levels (Fig. 6m). Moreover, the apoptosis level in chondrocytes was significantly decreased by exogenous administration of RGS12 (Fig. 6n).

## Discussion

Patients suffering from mitochondrial dysfunction, either due to a mitochondrial gene mutation or defects in nuclear genes encoding mitochondrial proteins, are reported to often present with short stature^34,35^. Here, we demonstrated that a critical function of RGS12 in mitochondria, most likely as a Tyr-phosphorylation enhancer, is essential for normal postnatal growth. This especially applies to mice at the age of 1 month, around pubertal growth. It was reported that the content and activity of mitochondria in osteoblasts are at a maximum level at 5 weeks of age, followed by a sharp drop and maintenance at a low level after 5 weeks during the life span of the rat from birth to old age^36,37^. Interestingly, we found that RGS12 KO mice showed the greatest impact on body weight, body length, and bone mass at ~4 weeks of age, accompanied by a significant decrease in mitochondrial number and activity. Mitochondrial dysfunction and damage contribute to cell apoptosis, decreased cell proliferation/viability rate and cell death^38^. More importantly, the damage responses in chondrocytes have been shown to destabilize the organization of the extracellular matrix, which then results in the expansion of the hypertrophic growth plate cartilage and reduced skeletal growth^38–40^. Similarly, we found that the loss of RGS12 leads to a disorder of mitochondria, apoptosis, and decreased cell proliferation/viability, which finally causes a delay in the process of chondrocyte maturation, showing an increased thickness in the growth plate and articular cartilage. Similar changes were also found in mice with mutant mitochondrial genes, which induced cell death at the cartilage-bone junction to cause a chondrodysplasia-like phenotype^35,41^.

Bone cells have been reported to increase the mitochondrial number during bone formation^42^. Mitochondrial dysfunction is thought to constitute the underlying mechanism of bone defects due to improper energy metabolism^43^. Our study reported that loss of RGS12 contributes to mitochondrial alterations, such as a decreased number of mitochondria, increased mitochondrial dysfunction, and enhanced apoptosis. Moreover, our study demonstrated that the apoptosis caused by RGS12 deletion is solely mitochondrial dependent since BAX, BCL-2, and CASPASE-9 were involved. A previous study showed that RGS12 is expressed in the nucleus and influences the cell cycle^44^. Here, we found that RGS12 also participates in the mitochondrial metabolism and regulates mitochondrial activity by binding to and activating mitochondrial OXPHOS (ATP5A). The proteins related to OXPHOS are located within the mitochondrial inner membrane and mainly affect three aspects: energy production, generation of ROS, and regulation of programmed cell death or apoptosis^45^. Accordingly, our results showed that RGS12 is involved in maintaining the mitochondrial membrane potential, energy production and ROS balance, which is critical for cell apoptosis and damage. Our experiments with isolated mitochondria showed that RGS12 is present in the mitochondria of control mice but not RGS12 KO mice, while overexpressed RGS12 restored RGS12 expression in mitochondria. Deletion or overexpression of RGS12 caused significant changes in mitochondrial function. Furthermore, we found that ATP5A was associated with RGS12 in mitochondria, and the changing trend of RGS12 expression was consistent in the cytoplasm and mitochondria, suggesting that RGS12 in mitochondria is critical for the maintenance of cell homeostasis. Thus, further studies will focus on local mitochondrial RGS12 therapy and the effects of conditional knockout of RGS12 in mitochondria.

RGS12 is a multidomain RGS protein that can regulate numerous molecular activities and signaling pathways^44,46^. Here, we identified a novel function by which RGS12 regulates mitochondrial proteins through Tyr phosphorylation. Willard MD et al. found that RGS12 associates with the tyrosine kinase TrkA to activate H-Ras, B-Raf, and MEK2 and further activate ERK^47^. Thus, one possible reason is that RGS12 could associate with tyrosine kinases such as TykA to enhance the Tyr phosphorylation of mitochondrial proteins such as ATP5A in mitochondria. However, due to mitochondrial enzyme complexity, the exact post-transcriptional regulatory mechanism still needs extensive study.

**Fig. 7:**
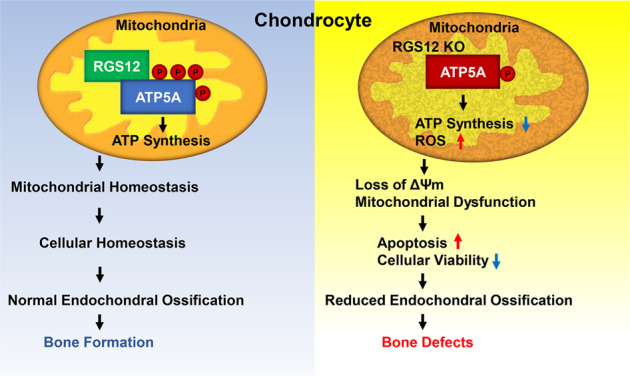
Proposed model of RGS12 regulation of bone formation via targeting of mitochondrial ATP5A. Under physiological conditions, RGS12 associates with ATP5A and activates p-Tyr of ATP5A to regulate the mitochondrial homeostasis, which maintains the normal endochondral ossification. The loss of RGS12 decreases the activity of ATP synthesis and increases the ROS level. Moreover, the loss of RGS12 contributes to low mitochondrial membrane potential (ΔΨm) and mitochondrial dysfunction. Finally, the mitochondrial dysfunction causes cell apoptosis and death which leads to reduced endochondral ossification and bone defects.

In summary, the findings of this study provide the first evidence that RGS12 contributes to the daily maintenance of mitochondrial homeostasis via ATP5A in chondrocytes (Fig. 7). Under physiological conditions, RGS12 activates p-Tyr of ATP5A to regulate mitochondrial homeostasis, which maintains normal endochondral ossification. However, the loss of RGS12 reduced the number and function of mitochondria and disturbed mitochondrial homeostasis, which further caused cell apoptosis and death and finally led to bone defects (Fig. 7). Therefore, this study provides genetic evidence that RGS12 may be a potential drug target for the treatment of bone defects caused by mitochondrial disorders.

## Materials and methods

### Cartilage-specific RGS12-knockout (KO) mice

To generate cartilage-specific RGS12 KO mice, RGS12^fl/fl^ mice were crossed with mice expressing cre recombinase under the control of the Col2-promotor (Col2-cre^+^). Col2-cre^+^, and the KO mice were littermates derived from the breeding of heterozygous animals. The animals were maintained under specific pathogen-free conditions. All animal studies were performed in accordance with institutional guidelines and with approval by the Institutional Animal Care and Use Committee (IACUC) of University of Pennsylvania.

### Growth curves

For phenotypic characterization, the weight of cartilage-specific RGS12 KO and control littermates was determined at postnatal days 1, 7, 14, 21, 28, and 56 of age.

### Cell culture and transfection

Cartilage of the knee and hip joints was harvested from 2-day-old mice followed by digestion with 0.2% collagenase (Thermo Scientific, US) for 4 h. Primary chondrocytes were grown in DMEM supplemented with 10% FBS and antibiotics that were changed every 3 days until the cells were confluent. Primary chondrocytes were seeded on 6-well plates at 3 × 10^5^ cells/well (~90–95% confluency). Cells were transfected on the following day with FuGENE HD Transfection Reagent (E2311, Promega Corporation, US). Cells were harvested at 48 h after transfection.

### Mitochondrial isolation

Mitochondria were isolated from cartilage using a mitochondrial isolation kit (Novus Biologicals, US) according to the manufacturer’s instructions. After washing with PBS, cartilage tissues were suspended and homogenized with extraction buffer and then centrifuged at 600 × *g* for 10 min at 4 °C. The supernatant was additionally centrifuged at 11,000 × *g* for 10 min to collect the final pellet, which consisted of mitochondrial protein. The concentration of mitochondrial protein was measured using a BCA Protein Assay Kit (Thermo Fisher Scientific, US).

### Plasmid construction

The full length of the RGS12 cDNA fragment (NM_173402.2) was cloned and inserted into the p3xFLAG-Myc-CMV-26 backbone. Full-length ATP5A cDNA (also known as Atp5f1a, NM_007505) was cloned and inserted into the pCMV6-Myc backbone.

### shRNA construction and virus production

Nontargeting RGS12 shRNAs were obtained from GeneCopoeia, Inc. Target sequences were as follows: Control shRNA: GCTTCGCGCCGTAGTCTTA; RGS12 shRNA1: CTAGGCAAGTCTAACTCTATT; RGS12 shRNA2: CCTGTCCATGATTAATAAAGG; and RGS12 shRNA3: AGTCTGCAACTGTGTCTGATGGCGAGTTG. Nontargeting ATP5A shRNAs were obtained from Santa Cruz Biotechnology (sc-60228-SH, Dallas, TX).

The 293 T packaging cell line was used for lentiviral amplification^48^. Briefly, viruses were collected at 72 h post-transfection. After passing through 0.45-µm filters, viruses were used to infect chondrocytes.

### Western blot

Cartilage or chondrocytes were homogenized with RIPA (radioimmunoprecipitation assay) buffer containing PIC (Protease Inhibitor Cocktails) (Sigma–Aldrich, US) on ice. Protein concentration was measured using BCA protein assay reagent (Thermo Fisher Scientific). Equal amounts of protein (30 μg) were denatured in SDS and separated in 10% SDS-PAGE gels. Proteins were transferred to PVDF membranes in transfer buffer containing 20% methanol. The membranes were blocked with 5% skim milk, incubated with primary antibody overnight at 4 °C and then incubated with a horseradish peroxidase (HRP)-conjugated secondary antibody (1:2,000, Jackson ImmunoResearch, PA) at room temperature for 1 h. α-TUBLIN (1:2,000, 11224-1-AP, Proteintech), β-ACTIN (1:2000, sc-47778, Santa Cruz) or COXIV (1:5000, 11242-1-AP, Proteintech) was used as the internal control. The following primary antibodies were used: anti-RGS12 (1:1,000, GW21317, Sigma–Aldrich), anti-ATP5A (1:1,000, 14676-1-AP, Proteintech), Total OXPHOS Rodent WB Antibody Cocktail (1:1000, Ab110413, Abcam), anti-p-Tyr (1:1000, Ab10321, Abcam), anti-BAX (1:2000, 50599-2-Ig, Proteintech), anti-BCL-2 (1:2000, 12789-1-AP, Proteintech), anti-pro-CASPASE-8 (1:2000, 4790, Cell Signaling Technology), anti-cleaved-CASPASE-8 (1:2000, 8592, Cell Signaling Technology), anti-pro-CASPASE-9 (1:2000, 9504, Cell Signaling Technology), and anti-cleaved-CASPASE-9 (1:2000, 9509, Cell Signaling Technology).

### Coimmunoprecipitation

ADTC5 cells (CRL-2846, ATCC) were lysed in NP-40 buffer supplemented with PIC and phenylmethylsulfonyl fluoride (Sigma–Aldrich, US). Briefly, lysates of equal amounts of protein were incubated at room temperature with primary antibodies for 1 h and then with protein A/G beads overnight, after which the beads were washed with PBST. Bound proteins were solubilized in loading buffer for western blot analysis.

### Histological assessment

Mouse knee joints were exposed by removing the overlying skin and were subsequently excised and decalcified with 0.5 M EDTA (pH = 8) for 14 days. A transverse cut was made when the bones were fully decalcified and processed for paraffin embedding. Tissues were cut into 8-µm sections and stained with hematoxylin/eosin or Safranin O/fast green to assess joint pathology.

### Immunofluorescence

Coverslips were incubated with primary antibodies against ATP5A (1:1,000, 14676-1-AP, Proteintech), RGS12 (1:1,000, GW21317, Sigma–Aldrich), cleaved-CASPASE-3 (1:2000, 9664, Cell Signaling Technology), and MitoTracker (1:100, M7514/M7512, Thermo Fisher Scientific) overnight at 4 °C. Cells were then incubated with the corresponding fluorochrome-conjugated secondary antibodies (diluted 1:500 in 1% BSA) for 1 h at room temperature. The relative fluorescence intensity was determined by comparing each intensity value to the average intensity of cells.

### Reactive oxygen species

The DCFDA (2′,7′-dichlorofluorescein diacetate) assay was used to analyze intracellular ROS levels. Chondrocytes were incubated with 10 μM DCFDA in serum-free medium at 37 °C for 30 min. Subsequently, ROS levels were immediately assessed using a fluorescence microscope (Leica, GER) to detect the mean fluorescence intensity (MFI) with an excitation wavelength of 488 nm and an emission wavelength of 525 nm.

### Mitochondrial membrane potential

The mitochondrial membrane potential (MMP) of chondrocytes was assessed using the fluorescent dye rhodamine 123. Chondrocytes were cultured in a 6-well plate and subsequently stained with 2 µM rhodamine 123 for 30 min at 37 °C in the dark. Cells were analyzed under an inverted fluorescence microscope. The fluorescence intensity was calculated with LAS Software (V4.3) (Leica Microsystems GmbH, Wetzlar, Germany).

### ADP, ATP, and ADP/ATP ratio

Intracellular ADP, ATP, and the ADP/ATP ratio were measured by an ADP/ATP Ratio Assay Kit (BioAssay Systems, US). Primary chondrocytes were seeded in white opaque microplates and treated with ATP/ADP reagent (BioAssay Systems, US). The ATP and ADP content in the supernatants was quantified using a highly sensitive luciferase-based technique. Luciferase activity was measured on a luminometer (BioTek, US) and compared with standards.

### Microcomputed tomography (micro-CT, μCT)

Two-dimensional images of the tibia by microcomputed tomography analysis were obtained. The parameters, bone mineral density (BMD; mg/cm), bone volume fraction (BV/TV; %), trabecular thickness (Tb.Th; mm), trabecular number (Tb.N; 1/mm), and trabecular separation (Tb.Sp; mm), were calculated using micro-CT software (Bone J, NIH).

### Quantitative real-time qPCR analysis

RNA from mouse cartilage or chondrocytes was isolated using TRIzol reagent (Thermo Fisher Scientific, USA) according to the manufacturer’s instructions. Then, 1 µg of RNA was reverse transcribed into cDNA using the Reverse Transcription Kit (TAKARA, Japan). Real-time PCR was performed with a reaction mixture containing primers, the cDNA template, and 2x SYBR Green qPCR Master Mix (Bimake, USA). The sequences of primers are shown in Supplementary Table S1.

### mtDNA copy number

The relative mtDNA copy number was determined by polymerase chain reaction (PCR). Total DNA from chondrocytes was extracted using a DNeasy Blood & Tissue Kit (QIAGEN, US) according to the manufacturer’s instructions. The mt-COX1 and mt-ND1 genes were amplified to determine the relative mtDNA copy number, with the 16 S gene used for normalization. The sequences of primers are shown in Supplementary Table S1.

### WST-1 viability assay

Primary chondrocytes were plated in triplicate in 96-well plates (1000 cells/well) in appropriate growth medium. The cell proliferation/viability assay was performed by following the WST-1 kit instructions (Sigma_Aldrich, USA). Briefly, at the indicated time points, cell medium was replaced with 90 µL fresh growth medium supplemented with 10 µL WST-1 reagents, followed by incubation at 37 °C for 1 h. OD absorbance values were measured at 490 nm using a 96-well plate reader (Bio-Rad, USA).

### Transmission electron microscopy

Primary chondrocytes from C57BL/6 mice were fixed overnight. Nanometer sections were cut and stained with 0.3% (w/v) lead citrate, and images were analyzed at the Electron Microscopy Core Facility.

For Immunoelectron microscopy (IEM), primary chondrocytes were cultured on plastic coverslips and fixed in 0.25% glutaraldehyde in PBS for 1 h at room temperature. After permeabilization in PBS containing 0.25% saponin for 30 min followed by blocking for 30 min in PBS containing 10% bovine serum albumin, the cells were exposed overnight to mouse primary antibodies in the blocking solution. The specimens were incubated with gold-conjugated goat anti-mouse-IgG (Abcam, 27241) in the blocking solution for 1 h at room temperature. The specimens were postfixed in 1% OsO_4_ containing 1.5% potassium ferrocyanide and were processed for electron microscopy similarly to the protocol for conventional electron microscopy.

### Statistical analysis

All data are expressed as the mean ± S.E.M. Statistical significance was determined by unpaired two-tailed Student’s *t*-test. An analysis of variance test was first performed to compare the mean values between groups, and the Student–Newman–Keuls test was used to compare the mean values between two conditions with GraphPad software 7.0 (San Diego, CA, US). In all cases, *P*-values less than 0.05 were considered significant.

## Supplementary information

Supplementary Information

## Supplementary information

**Supplementary Information** accompanies the paper at (10.1038/s41421-020-00190-w).
