# Supplementary material for: RGS12 is required for the maintenance of mitochondrial function during skeletal development
Source: Cell Discov. 2020 Sep 1;6:59. doi: 10.1038/s41421-020-00190-w (PMC7459111; doi:10.1038/s41421-020-00190-w)
Supplement: Supplementary file 1 — Supplementary Information [file 41421_2020_190_MOESM1_ESM.pdf]

Supplementary Figures and Figure legends

Supplementary Fig. S1

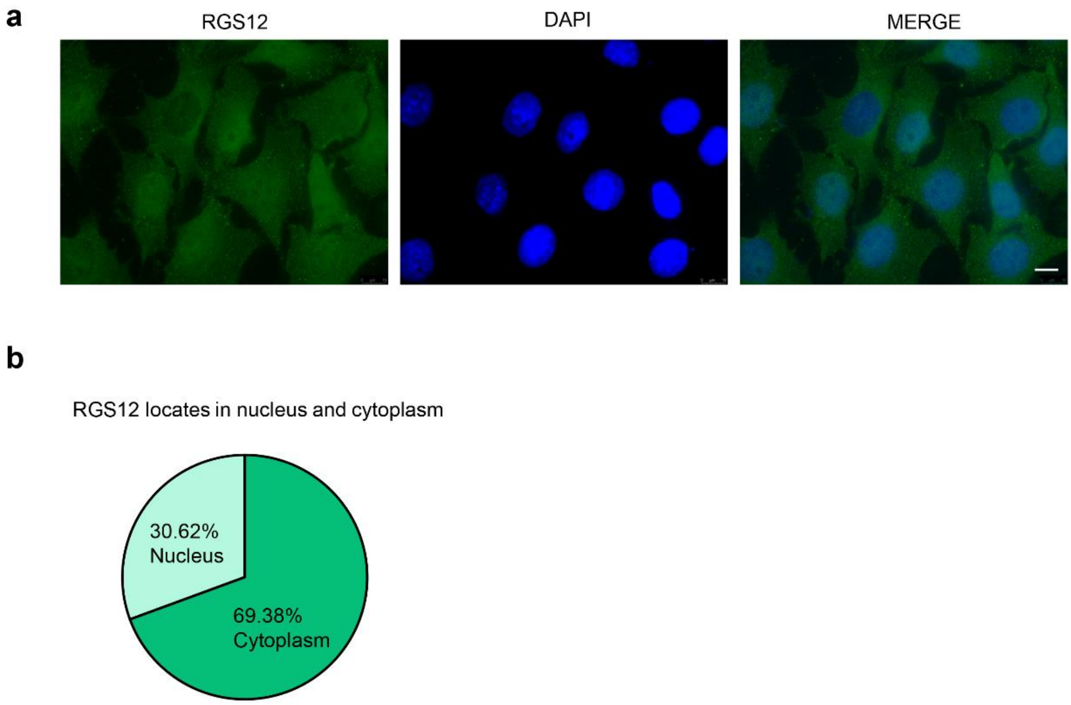

Supplementary Fig. S1 RGS12 locates in chondrocytic cytoplasm and nucleus.

(**a**) Immunofluorescence showing that RGS12 locates in cytoplasm and nucleus. Scale bar, 10µm. Representative individual and overlaid images are shown. (**b**) Relative RGS12 intensity is in (**a**). Note that RGS12 mostly expresses in cytoplasm in chondrocyte.

Supplementary Fig. S2

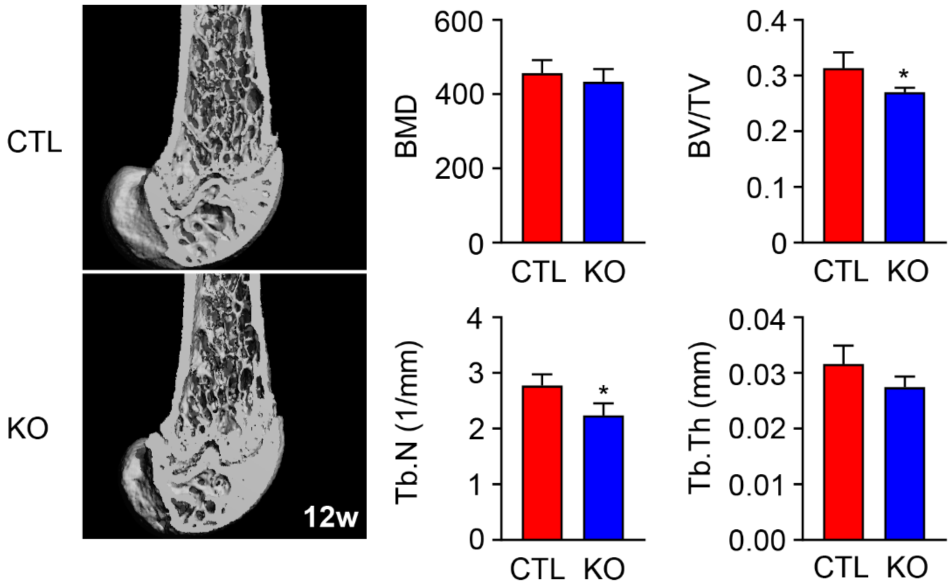

Supplementary Fig. S2 Micro-CT images of the femoral metaphyseal region of 12-week-old mice from the CTL and RGS12 KO groups. Histograms represent the trabecular structural parameters: bone mineral density (BMD), trabecular bone volume/tissue volume (BV/TV), trabecular number (Tb.N), trabecular thickness (Tb.Th). Note: At the age of 12 weeks, only BV/TV and trabecular number show a significant difference between RGS12 KO and CTL, suggesting an early difference in bone growth in cartilage specific RGS12 KO mice. Data are presented as means ± SEM. n = 5. * $P < 0.05$.

Supplementary Fig. S3

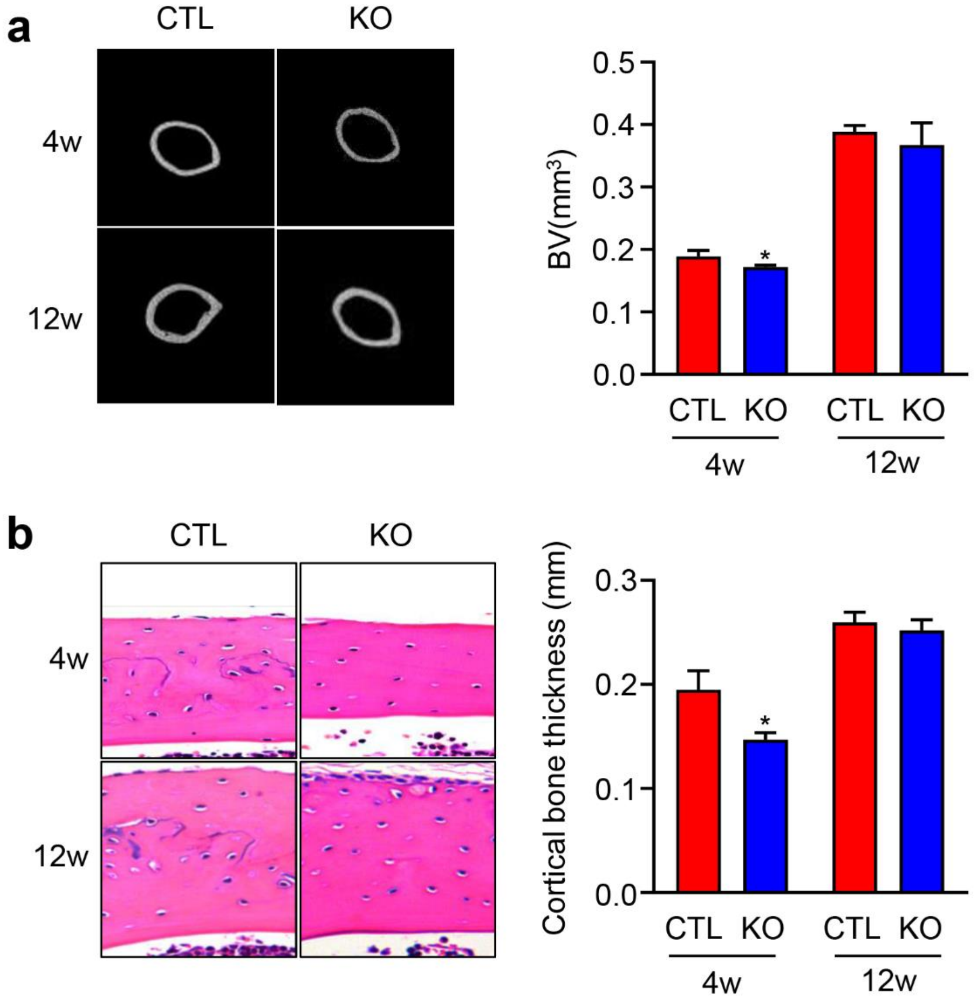

Supplementary Fig. S3 Knockout of RGS12 causes reduction in cortical bone volume and thickness in 4-week-old mice.

(**a**) Representative microCT images of cortical bone at the femoral diaphysis of 4-week and 12-week-old mice from the CTL and RGS12 KO groups. Histograms represent the quantitative analyses of cortical bone volume (BV) in femora.

(**b**) Histologic sections of cortical bone from femoral mid-diaphysis of 4-week-old and 12-week-old CTL and RGS12 KO mice (left). Histomorphometric analysis (right). Data are means ± S.E.M. *$P < 0.05$.

Supplementary Fig. S4

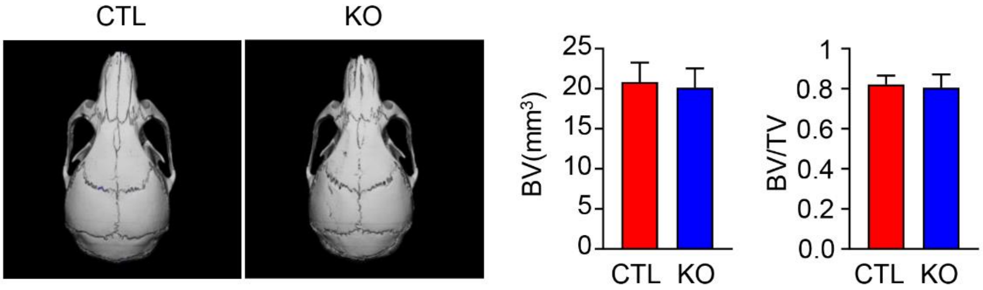

Supplementary Fig. S4 Comparison of cranial morphologies and calvarial bone mass among 4-week-old RGS12 KO mice. Measurement of calvarial bone mass showed that RGS12 KO calvarias exhibited no significant change on BV or BV/TV.

Supplementary Fig. S5

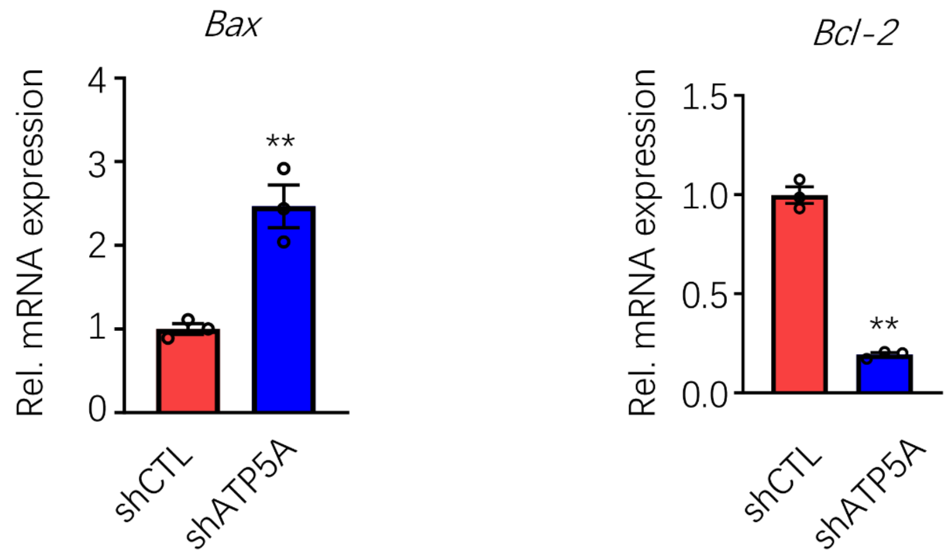

Supplementary Fig. S5 Knockdown of ATP5A leads to cell apoptosis. Expression levels of *Bax* and *Bcl-2* in Control shRNA (shCTL) and ATP5 shRNA (shATP5A) chondrocytes were determined by quantitative PCR. Data are means ± S.E.M. $^{**}P < 0.01$.

Table S1. Mouse Primers Used for PCR Analysis

Gene		Primer Sequence	Ta(ºC)
Rgs12	Forward	CAGAGTACCCTGCCGAGAAG	60
	Reverse	AGTCTGGGTCCACCATGAAC	
Atp5a	Forward	GCCCTCGGTAATGCTATTGA	60
	Reverse	GCAATCGATGTTTTCCCAGT	
Bax	Forward	TGCAGAGGATGATTGCTGAC	60
	Reverse	GATCAGCTCGGGCACTTTAG	
Bcl-2	Forward	CTGGCATCTTCTCCTTCCAG	60
	Reverse	GACGGTAGCGACGAGAGAAG	
Col10a1	*Forward*	GCAGCATTACGACCCAAGAT	60
	Reverse	TCTGTGAGCTCCATGATTGC	
Sox9	*Forward*	CGACTACGCTGACCATCAGA	60
	Reverse	AGACTGGTTGTTCCCAGTGC	
Acan	*Forward*	TGGCTTCTGGAGACAGGACT	60
	Reverse	TTCTGCTGTCTGGGTCTCCT	
Gapdh	*Forward*	AGGTCGGTGTGAACGGATTTG	60
	Reverse	TGTAGACCATGTAGTTGAGGTCA	
16S	*Forward*	GGGATAACAGCGCAATCCTA	60
	Reverse	GATTGCTCCGGTCTGAACTC	
mtND1	*Forward*	CCTTCGACCTGACAGAAGGA	60
	Reverse	GATGCTCGGATCCATAGGAA	
mtCOX1	*Forward*	GCCTTTCAGGAATACCACGA	60
	Reverse	AGGTTGGTTCCTCGAATGTG	
